# Determinants of Survival in Malignant Pleural Mesothelioma: A Surveillance, Epidemiology, and End Results (SEER) Study of 14,228 Patients

**DOI:** 10.1371/journal.pone.0145039

**Published:** 2015-12-14

**Authors:** Emanuela Taioli, Andrea S. Wolf, Marlene Camacho-Rivera, Andrew Kaufman, Dong-Seok Lee, Daniel Nicastri, Kenneth Rosenzweig, Raja M. Flores

**Affiliations:** 1 Department of Thoracic Surgery, Mount Sinai Medical Center, One Gustave L. Levy Place, Box 1023, New York, New York, 10029, United States of America; 2 Department of Population Health Science and Policy and Institute for Translational Epidemiology, Icahn School of Medicine at Mount Sinai, One Gustave L. Levy Place, Box 1077, New York, New York, 10029, United States of America; 3 Department of Community Health and Social Medicine, Sophie Davis School of Biomedical Education, City College of New York, New York, 10031, United States of America; 4 Department of Oncology, Mount Sinai Medical Center, One Gustave L. Levy Place, Box 1023, New York, New York, 10029, United States of America; Taipei Medical University, TAIWAN

## Abstract

**Introduction:**

Left untreated, malignant pleural mesothelioma (MPM) is associated with uniformly poor prognosis. Better survival has been reported with surgery-based multimodality therapy, but to date, no trial has demonstrated survival benefit of surgery over other therapies. We evaluated whether cancer-directed surgery influenced survival independently from other predictors in a large population-based dataset.

**Methods:**

The SEER database was explored from 1973 to 2009 to identify all cases of pathologically-proven MPM. Age, sex, race, year of diagnosis, histology stage, cancer-directed surgery, radiation, and vital status were analyzed. The association between prognostic factors and survival was estimated using Cox regression and propensity matched analysis.

**Results:**

There were 14,228 patients with pathologic diagnosis of MPM. On multivariable analysis, female gender, younger age, early stage, and treatment with surgery were independent predictors of longer survival. In comparison to no treatment, surgery alone was associated with significant improvement in survival [adjusted hazard ratio (adj HR) 0.64 (0.61–0.67)], but not radiation [adj HR 1.15 (1.08–1.23)]. Surgery and radiation combined had similar survival as surgery alone [adj HR 0.69 (0.64–0.76)]. Results were similar when cases diagnosed between 1973 and 1999 were compared to cases diagnosed between 2000 and 2009.

**Conclusions:**

Despite developments in surgical and radiation techniques, the prognosis for MPM patients has not improved over the past 4 decades. Cancer-directed surgery is independently associated with better survival, suggesting that multimodal surgery-based therapy can benefit these patients. Further research in adjuvant treatment is necessary to improve prognosis in this challenging disease.

## Introduction

Mesothelioma is a rare but deadly cancer that has been linked to occupational and environmental asbestos exposure. The incidence of mesothelioma has increased greatly starting from the seventies, when the effects of past exposure to asbestos became evident, and still shows no signs of decline in the US, despite the fact that asbestos use has been banned several decades ago [1]. The diagnosis of mesothelioma often occurs when the disease is already at an advanced stage, and life expectancy is usually limited to few months. In recent years, several therapeutic approaches have been attempted with the hope to extend survival, including surgery, radiation, surgery combined with radiation, chemotherapy in various combinations with radiotherapy and surgery. A recent meta-analysis comparing survival after extra-pleural pneumonectomy and pleurectomy decortication [2] suggests that less invasive surgical approaches, such as pleurectomy/decortication are associated with prolonged survival. A randomized controlled trial conducted in the UK to assess survival, complications, and quality of life after extra-pleural pneumonectomy [3] suggested that the extensive surgical approach did not offer any survival advantage over chemotherapy alone.

Despite the many studies published so far, there is no agreement on which is the optimal therapeutic strategy that would obtain the longest survival of mesothelioma patients. Because mesothelioma is a rare disease, single institutions rarely collect a large enough number of cases to conduct outcome studies on the effects of the various therapeutic modalities. The Surveillance, Epidemiology and End Results (SEER) data base includes a large population based sample of unselected cancer patients for which information on tumor characteristics at diagnosis, type of therapy and outcome is available. We have analyzed the mesothelioma cases present within SEER, to study the effect of therapy and other prognostic factors on survival.

## Methods

The SEER database was explored from 1973 to 2009, and all cases identified as mesothelioma within the site recode ICD-O-3 variable by ICD-O-3 morphology were extracted. Only patients with pathologically proven malignant mesothelioma of pleura and lung were included. Exclusion criteria included age below 18 years old, all postmortem cases, non-microscopically confirmed cases (for which no pathology confirmation of the diagnosis was available), and any case without survival time in the database (n = 1,077). Malignant mesothelioma of other sites (retroperitoneal, peritoneal, genital, heart, mediastinum, soft tissue, digestive, other, and unknown primary site) were also excluded.

The SEER 09 registry included cases from Atlanta, Connecticut, Detroit, Hawaii, Iowa, New Mexico, San Francisco-Oakland, Seattle-Puget Sound, and Utah from 1973–2004. The SEER 13 registry included cases from Los Angeles, San Jose-Monterrey, rural Georgia, and Alaska in addition to SEER 09 cases. The SEER 17 registry included cases from Greater California, Kentucky, Louisiana, and New Jersey from 2000 to 2004. The SEER 18 registry included cases from Greater Georgia from 2000 and on, with the exception of adjustments for the areas impacted by Hurricanes Katrina and Rita. SEER is a publicly available resource containing anonymous information, and as such data extracted from SEER was deemed “non-human study” by the North Shore LIJ IRB committee.

### Definition of Staging

Localized: Invasive tumor confined to pleura; ipsilateral parietal and/or visceral pleura; mesothelioma with nodules beneath the visceral pleural surface; and localized, not otherwise specified. *Regional*: Extension to adjacent organs/structure: adjacent connective tissue, pericardium, endothoracic fascia, diaphragm; mesothelioma nodules that have broken through the visceral pleural surface to the lung surface, lung involvement not otherwise specified; extension to adjacent organs such as the chest wall, ribs, heart muscle, mediastinal organs and tissues; mesothelioma with malignant pleural fluid/effusion; regional ipsilateral lymph nodes; and regional not otherwise specified.

Distant: Contralateral pleura and lung, extension to intraabdominal organs, cervical tissues, peritoneum, metastasis; further contiguous extension; unknown if extension or metastasis; and distant lymph nodes.

### Definition of Cancer-directed surgery

For cases diagnosed after 1998, they were identified as having received cancer-directed surgery if given any of the following codes for the “Rx Summ-Surg Prim Site” variable: 30 = simple/partial surgical removal of primary site; 40 = total surgical removal of primary site, enucleation; 50 = “debulking”; 60 = radical surgery which included partial or total removal of the primary site with a resection in the continuity (partial or total removal) with other organs.

For cases prior to 1998, cases were identified as having received cancer-directed surgery if given any of the following codes for the “Site Specific Surgery” variable: (10 = Local surgical excision or destruction of lesion; 20 = Partial/wedge/segmental resection; 30, 40 = Lobectomy/ bilobectomy with/without dissection of lymph nodes; 50 = Complete/total/standard pneumonectomy, pneumonectomy, NOS; 60 = Radical pneumonectomy plus dissection of mediastinal lymph nodes; 70 = Extended radical pneumonectomy with diaphragm plus lymph nodes; 90 = Resection of lung, NOS; surgery, NOS.

For all cases, the code 00 (which indicated “no surgical procedure had been performed”) and the codes for other types of surgery (codes 01 = Incisional, needle, or aspiration biopsy of other than primary site; 02 = Incisional, needle, or aspiration biopsy of primary site; 03 = Exploratory only (no biopsy); 04 = Bypass surgery, -ostomy only (no biopsy); 05 = Exploratory only and incisional, needle or aspiration biopsy of primary site or other sites) were used to categorize patients who did not undergo cancer-directed surgery.

### Statistical analysis

Variables analyzed include age at diagnosis, sex, race, year of diagnosis, vital status, stage, surgery, and radiation. Overall survival was defined as the time between the initial diagnosis date and either date of death or last follow-up. Univariate analyses of survival in relation to patient’s demographics and tumor characteristics were conducted by the Ederer II method. The independent contribution to survival of several prognostic factors was analyzed with multivariate regression methods based on the Cox proportional hazards model. A propensity analysis for the association of surgery with survival, matched on sex, age, and stage was also conducted. All analyses were performed using SAS version 9.2 (Cary, NC, USA).

## Results

There were over 14,000 cases of malignant pleural mesothelioma (MPM) in the SEER data set. A description of the population is reported in Table 1. The majority of the patients were white, and roughly three quarters of the cases were males; median age at diagnosis was 62 years. More than half of the cases were diagnosed with distant metastases. Only 23% of the cases received cancer-directed surgery, and 13% received radiation therapy. Localized cases are more likely to be treated with radiation only, while regional cases with surgery only or in combination with chemotherapy. Distant cases are less likely to receive surgery or radiotherapy; probably other palliative care is used, which is not collected by SEER. The median overall survival was 7 months,; the large part of the patients (91%) was deceased at the end of follow-up.

**Table 1:** Patient, Disease, and Treatment Characteristics (*n* = 14228), SEER (Surveillance, Epidemiology, and End Results).

Variable	Categories	Total
**Registry**	SEER 9	7994 (56%)
	SEER 13 (> 1992)	1623 (11%)
	SEER 17 (>2000)	4227 (30%)
	SEER 18	384 (3%)
**Sex**	Male	11032 (78%)
	Female	3196 (22%)
**Race**	White	13046 (92%)
	Black	688 (5%)
	Other	494 (3%)
**Age (yr)**	18–49	910 (6%)
	50–59	1795 (13%)
	60–69	3421 (24%)
	70–79	4875 (34%)
	80+	3227 (23%)
**Diagnosis year**	1973–1989	2695 (19%)
	1990–1994	1514 (11%)
	1995–1999	1764 (12%)
	2000–2004	4048 (28%)
	2005–2009	4207 (30%)
**Overall Stage**	Localized	1572 (11%)
	Regional	2355 (16%)
	Distant	8367 (59%)
	Unknown	1934 (14%)
**Histology**	Fibrous	1096 (8%)
	Epithelial	3292 (23%)
	Biphasic	650 (4%)
	Mesothelioma, NOS	9190 (65%)
**Cancer-directed surgery**	No	10921 (77%)
	Yes	3307 (23%)
**Type of cancer-directed surgery**	Partial resection	542 (16.3%)
	Lobectomy	1232 (37.3%)
	Pneumonectomy	808 (24.5%)
	Resection NOS	725 (21.9%)
**Radiation**	No	12050 (85%)
	Yes	1805 (13%)
	Unknown	373 (2%)
**Vital Status**	Alive	1326 (9%)
	Dead	12902 (91%)
**Survival in months**	Median	7
	1973–1989	8
	1990–1994	7
	1995–1999	7
	2000–2004	7
	2005–2009	6

**Fig 1:**
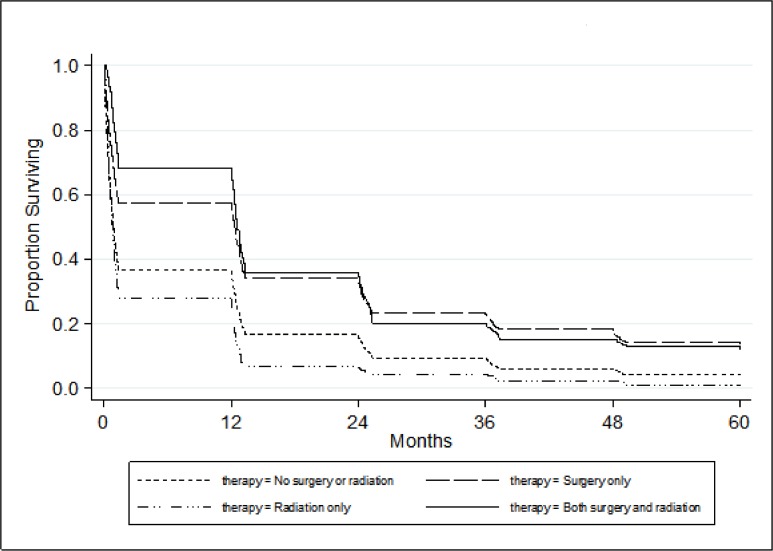
Survival according to type of treatment (SEER database).

Predictors of survival: at univariate analysis, survival was longer at younger ages, in females, in cases that were diagnosed at early stages, in patients treated with surgery or a combination of surgery and radiation (Fig 1). At multivariate analysis (Table 2), independent significant predictors of survival were: being female [adjusted Hazard Ratio (adjHR): 0.79 (95% CI: 0.75–0.82)], disease stage [adjHR for distant versus local disease: 1.4 (95% CI: 1.31–1.49)], and age [adjHR: 1.02 (95% CI: 1.02–1.02) with increasing age]. Survival was also improved in the most recent calendar year of diagnosis (adjHR: 0.81 (95%CI: 0.77–0.86) for patients diagnosed in 2005–2009 versus patients diagnosed in 1973–1989). Epithelial histology was associated with best survival in comparison to the other histologic types.

**Table 2:** Association between Patient and Disease Characteristics and Survival.

Variable	Category	Adjusted HR (95% CI) *
Sex	Male	1 (Ref)
	Female	0.79 (0.75–0.82)
Race	White	1 (ref)
	Black	1.07 (0.98–1.16)
	Other	0.98 (0.89–1.08)
Age (years)	continuous	1.02 (1.02–1.02)
Stage	Localized	1 (ref)
	Regional	1.35 (1.25–1.44)
	Distant	1.40 (1.31–1.49)
Histology	Epithelial	1 (ref)
	Fibrous	1.54 (1.43–1.66)
	Biphasic	1.46 (1.33–1.60)
	Meso NOS	1.18 (1.13–1.24)
Diagnosis year	1973–1989	1 (ref)
	1990–1994	0.92 (0.86–0.98)
	1995–1999	0.88 (0.82–0.93)
	2000–2004	0.87 (0.83–0.92)
	2005–2009	0.81 (0.77–0.86)
Therapy	No radiation or surgery	1 (ref)
	Radiation only	1.15 (1.08–1.23)
	Surgery only	0.64 (0.61–0.67)
	Radiation and surgery	0.69 (0.64–0.76)

*Adjusted for all other variables in the table

In comparison to no surgery/radiation treatment, surgery alone was associated with significant improvement in survival [adj HR 0.64 (95% CI: 0.61–0.67)], while radiation did not improve survival [adj HR 1.15 (95% CI: 1.08–1.23)]. Surgery and radiation combined was associated with similar survival as surgery alone [adj HR 0.69 (95% CI: 0.64–0.76)]. The median survival of the group not receiving radiotherapy or surgery was 6.5 months, very similar to the median survival of the group treated with radiotherapy (7.5 months), while median survival in the surgical group was 14.5 months, and in those receiving both radiation and surgery was 13 months. A stratified analysis according to calendar year of diagnosis shows that cases diagnosed earlier on are also experiencing worse survival than cases diagnosed in more recent times (Table 3). In patients diagnosed between 1973 and 1999, the adj HR for radiation was 1.14 (95% CI: 1.05–1.23), for surgery 0.63 (95% CI: 0.59–0.68), for surgery plus radiation 0.75 (95% CI: 0.66–0.84); similar results were obtained in patients diagnosed between 2000 and 2009.

**Table 3:** Effect of therapy on survival according to period of diagnosis.

	Adjusted HR (95% CI) *	
Therapy	1973–1999	2000–2009
No radiation or surgery	1 (Ref)	1 (Ref)
Radiation only	1.14 (1.05–1.23)	1.26 (1.14–1.41)
Surgery only	0.63 (0.59–0.68)	0.68 (0.64–0.73)
Radiation and surgery	0.75 (0.66–0.84)	0.65 (0.57–0.73)

*Adjusted for sex, race, age, and stage

To confirm that the relative advantage of surgery on survival is not due to differences in patient characteristics among those who received surgery compared to other treatment categories, a propensity score analysis was conducted where patients were propensity balanced based on sex, stage, and age using the nearest neighbor method. The effect of surgery on survival in the raw data set and after adjustment using the propensity score method was not significantly different from one another (HR = 0.64; 95% CI 0.61–0.67 and matched HR = 0.68; 95% CI 0.66–0.72 respectively).

## Discussion

The present analysis of a large population based cancer data set suggests that cancer-directed surgery is independently associated with better survival, alone or in combination with radiotherapy, suggesting that multimodal surgery-based therapy can benefit MPM patients. The median survival of the group treated with surgery was double the value observed in those not receiving radiotherapy or surgery, or among those treated with radiotherapy. These differences are meaningful in a disease that has a very quick evolution and extremely short survival rate, and are very similar to what reported by others [4]. One aspect that needs to be considered is that the SEER program includes data from both general hospitals and highly specialized cancer centers, and the surgical techniques used may greatly differ between these two hospital settings. centers. However, the improved survival with multi-modal therapy reported here confirms results of individual studies conducted in Europe [4] or in the US [5] on smaller series. Bovolato et al showed a statistically significant improvement in patients who underwent a surgical approach versus those who were non-surgically treated [4]. Kapeles et al [5]suggests that patients treated with trimodality therapy have a significantly improved survival, but the result is not confirmed by others [6]. The Kapeles study however does not disentangle the effects of each individual procedure (surgery versus radiation versus chemotherapy); other predictors of survival were gender and age, similar to what we report here.

Radiation for MPM has been shown to be effective in trials conducted at specialized centers [7] suggesting that such approach should be conducted by experts in the field. Results from our study indicate that surgery is the main determinant of survival, alone or in combination with radiation.

The present study follows a previously published SEER analysis from our group [8] on a smaller sample of patients (n = 5937), on the predictors of undergoing surgery. The study found that age, race and stage were main factors associated with the surgical approach, and provided the first evidence that surgery was associated with improved survival. However, the paper did not compare survival according to the different treatment approaches as we have done here.

The results we present have several limitations: patients are usually selected for surgery according to a combination of clinical factors such performance status, pulmonary and cardiac function, and comorbid conditions, which are not recorded in the SEER data base, and could contribute to survival. Therefore, although we conducted a propensity analysis taking into account age, sex and stage, we could not consider other important factors such as comorbidities.

Another limitation is that the SEER data base does not record whether patients were treated with curative-intent versus palliative-intent, nor if patients received chemotherapy, what chemotherapeutic agents were used and in what dosage or scheme. The contribution of chemotherapy alone on MPM survival seems to be modest: a recent review of the published randomized clinical trials comparing medical treatment shows that only 10 RCT were conducted comparing two chemo-therapy regimens, and only the 2 involving platinum-based compounds showed a statistically significant improvement in survival, in the order of 2–3 months difference [9]. A previously published study [4] suggests that MPM surgical approach improves survival over non-surgical approach (including radiation and chemotherapy).

Another limitation of the large SEER data base is that radiotherapy details are not well-documented, for example the radiation dose or radiation fields cannot be determined. Another missing information is the sequence of the various therapeutic approaches.

However, this collection of MPM is one of the largest published in the literature, and is informative for additional observational and experimental studies comparing treatment strategies for MPM. Despite developments in surgical and radiation techniques, the prognosis for pleural mesothelioma patients has not improved over the past 4 decades. A possible exception is represented by BAP1 mutated MPM patients [10]. In these patients, MPM seems to progress very slowly and surgery might be particularly indicated as main treatment. Further research on the impact of adjuvant treatment, and of new approaches such as gene therapy and immunotherapy, alone or in combination with surgery, is necessary to improve prognosis in this challenging disease [11].
